# Diagnostic analysis of the highly complex *OPN1LW/OPN1MW* gene cluster using long-read sequencing and MLPA

**DOI:** 10.1038/s41525-022-00334-9

**Published:** 2022-11-09

**Authors:** Lonneke Haer-Wigman, Amber den Ouden, Maria M. van Genderen, Hester Y. Kroes, Joke Verheij, Dzenita Smailhodzic, Attje S. Hoekstra, Raymon Vijzelaar, Jan Blom, Ronny Derks, Menno Tjon-Pon-Fong, Helger G. Yntema, Marcel R. Nelen, Lisenka E.L.M. Vissers, Dorien Lugtenberg, Kornelia Neveling

**Affiliations:** 1 Department of Human Genetics, Radboud University Medical Center, Nijmegen, The Netherlands; 2 Donders Institute for Brain, Cognition and Behaviour, Radboud University Medical Center, Nijmegen, The Netherlands; 3 Bartiméus Diagnostic Center for complex visual disorders, Zeist, The Netherlands; 4 Department of Ophthalmology, University Medical Centre Utrecht, Utrecht, The Netherlands; 5 Department of Medical Genetics, University Medical Center Utrecht, Utrecht, The Netherlands; 6 Department of Genetics, University Medical Center Groningen, University of Groningen, Groningen, The Netherlands; 7 The Rotterdam Eye Hospital, Rotterdam, 3011 BH The Netherlands; 8 MRC Holland b v, Amsterdam, The Netherlands

**Keywords:** Molecular medicine, Medical genetics

## Abstract

Pathogenic variants in the *OPN1LW/OPN1MW* gene cluster are causal for a range of mild to severe visual impairments with color deficiencies. The widely utilized short-read next-generation sequencing (NGS) is inappropriate for the analysis of the *OPN1LW/OPN1MW* gene cluster and many patients with pathogenic variants stay underdiagnosed. A diagnostic genetic assay was developed for the *OPN1LW/OPN1MW* gene cluster, consisting of copy number analysis via multiplex ligation-dependent probe amplification and sequence analysis via long-read circular consensus sequencing. Performance was determined on 50 clinical samples referred for genetic confirmation of the clinical diagnosis (*n* = 43) or carrier status analysis (*n* = 7). A broad range of pathogenic haplotypes were detected, including deletions, hybrid genes, single variants and combinations of variants. The developed genetic assay for the *OPN1LW/OPN1MW* gene cluster is a diagnostic test that can detect both structural and nucleotide variants with a straightforward analysis, improving diagnostic care of patients with visual impairment.

## Introduction

Color vision and vision in non-dimmed light is regulated by the cones in the retina. Humans have three types of cone opsins with different relative spectral sensitivities; the short-wavelength, middle-wavelength, and long-wavelength sensitive opsins have an optimal spectral sensitivity of the blue, green and red light spectrum, respectively. Each cone in the retina only expresses one type of cone opsin and is therefore sensitive to one of the three spectra.

**Fig. 1:**
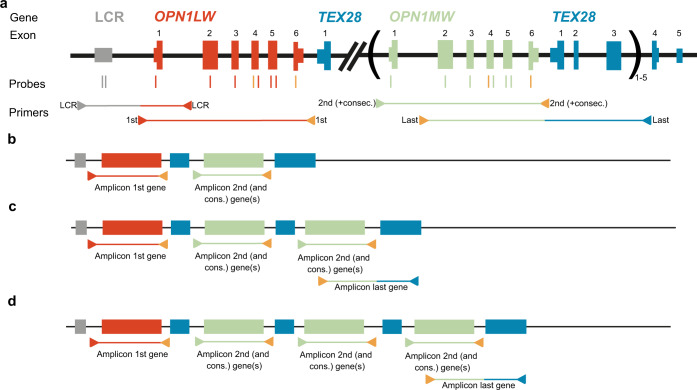
Schematic overview of the *OPN1LW*/*OPN1MW* gene cluster and the position of the MLPA probes and sequencing primers. **a** The *OPN1LW*/*OPN1MW* gene cluster consist of the LCR, one *OPN1LW* gene and one to five *OPN1MW* genes. Downstream of the OPN1 cluster, the *TEX28* gene is located. MLPA probes are depicted as bars, probes specific for the LCR, specific for *OPN1LW* or specific OPN1MW are depicted in gray, red or green respectively and probes targeting both *OPN1LW* and *OPN1MW* are depicted in orange. Sequencing primers are depicted as arrows, forward primers point to the right and reverse primers point to the left. Primer(s) binding in the LCR, *OPN1LW*, *OPN1MW* or *TEX28* are depicted in gray, red, green or blue respectively. Primers that can bind to both *OPN1LW* and *OPN1MW* are depicted in orange. **b** If the *OPN1LW/OPN1MW* gene cluster has two genes, *OPN1LW* will be sequenced with the amplicon for the first gene and *OPN1MW* will be sequenced with the amplicon for the second and consecutive genes. **c** If the *OPN1LW/OPN1MW* gene cluster has three genes, *OPN1LW* will be sequenced with the amplicon for the first gene and both *OPN1MW* genes will be sequenced with the amplicon for the second and consecutive genes. An additional amplicon was developed for the last gene, in this case the third gene. When the genetic composition of this last third clinically irrelevant gene is determined, the genetic composition of the clinically relevant second gene can be deduced. **d** If the *OPN1LW/OPN1MW* gene cluster has four genes, *OPN1LW* will be sequenced with the amplicon for the first gene and all three copies of *OPN1MW* will be sequenced with the amplicon for the second and consecutive genes. In this situation amplification of the last gene is not useful, as one is unable to differentiate between the clinically relevant second and clinically irrelevant third gene in the cluster.

The middle-wavelength and the long-wavelength sensitive opsin are encoded by *OPN1MW* [MIM: 300821] and *OPN1LW* [MIM: 300822], respectively. *OPN1LW* and *OPN1MW* are in close proximity located on the X-chromosome and share more than 98% sequence homology^1,2^. The so-called *OPN1LW*/*OPN1MW* gene cluster generally consists of a single *OPN1LW* and up to five *OPN1MW* genes located head-to-tail in tandem (Fig. 1a)^2,3^. Approximately 3.5 kilobases (kb) upstream of *OPN1LW* the locus control region (LCR) is located (Fig. 1a), which ensures the expression of either the first or second opsin gene downstream of the LCR^4–6^.

Variants in the *OPN1LW*/*OPN1MW* gene cluster cause a range of mild to severe X-linked color vision deficiencies^7–9^. Red or green color deficiency (protanopia [MIM: 303900] or deuteranopia [MIM: 303800], respectively) with normal visual acuity and cone electroretinogram (ERG), affects approximately 1 in 12 males and 1 in 200 females. Bornholm eye disease (BED [MIM: 300843]), a more severe visual impairment, is associated with high myopia, mildly to moderately reduced visual acuity, and reduced red and green cone ERG, in addition to protanopia or deuteranopia^10^. Patients with blue cone monochromacy (BCM [MIM: 303700]) only have perception of the blue light spectrum, combined with congenital nystagmus, photophobia, high myopia, absent red and green cone ERG, and severely reduced visual acuity^11^.

Different types of pathogenic variants have been detected in the *OPN1LW*/*OPN1MW* gene cluster^12–14^. Due to the high homology and the close proximity of *OPN1LW* and *OPN1MW*, rearrangements occur frequently^1,15–17^. Nonhomologous recombination can cause the formation of hybrid opsin genes that encode a functional opsin protein with an optimal spectral sensitivity for the red or green light spectrum^1,9,18^. Another frequent structural variant is a deletion of the LCR causing BCM^4,15,19^. Pathogenic single nucleotide variants have also been described, e.g., the c.607C > T p.(Cys203Arg) variant which is frequently detected in patients with BCM^19,20^. Moreover, exon 3 in both *OPN1LW* and *OPN1MW* has a region encompassing eight nucleotide variants encoding seven amino acids (c.453G > A p.(Arg151Arg), c.457C > A p.(Leu153Met), c.465G > A p.(Val155Val), c.[511G > A;513G > T] p.(Val171Ile), c.521C > T p.(Ala174Val), c.532 A > G p.(Ile178Val), c.538G > T p.(Ser180Ala)) each occurring frequently in the population, however, specific rare combinations of these variants are pathogenic as they induce incorrect splicing^21–23^. For example, the LIAVA combination (a combination is denoted by taking the one letter amino acid abbreviations of the five amino acids at positions 153, 171, 174, 178 and 180) in *OPN1LW* and the MVVVA combination in *OPN1MW* is causal for BED^21,23,24^.

Only a few diagnostic genetic tests are currently available to analyze the *OPN1LW*/*OPN1MW* gene cluster^25^. Most tests do not solely target the cluster, but the *OPN1LW/OPN1MW* genes are tested as part of broader gene panels and analyzed by short-read next-generation sequencing (NGS)^25^. Short-read sequencing renders reads of 100-400 base pairs (bp) in length, which are too short to differentiate between the highly homologous *OPN1LW* and *OPN1MW* gene (Supplementary Fig. 1)^26,27^. Moreover, none of the available tests combine copy number and sequence analysis, which is needed to detect the complete spectrum of causal variants^25^. Due to the unavailability of complete genetic testing of the *OPN1LW/OPN1MW* opsin gene cluster many patients with pathogenic variants stay underdiagnosed. Probably, genetic underdiagnosis contributes to the lack of awareness by clinicians to recognize retinal disease caused by dysfunctional opsin genes.

The aim of this study is to facilitate and improve diagnostic care of patients with visual impairment with color deficiencies by developing a genetic assay for the analysis of the *OPN1LW*/*OPN1MW* gene cluster combining copy number analysis using multiplex ligation-dependent probe amplification (MLPA) and sequencing analysis using long-read circular consensus sequencing.

## Results

### Performance of the opsin MLPA on control samples

MLPA is a technique to quantify copy numbers of pre-selected targets, in this case, genomic targets within the opsin locus. Each target renders a fluorescent signal and comparison of these signals, by calculation of a ratio between a sample and a single or a set of reference samples with known copy number(s), is used to determine the copy number of the sample. As there is no publicly accessible reference sample yet with described *OPN1LW* and *OPN1MW* copy numbers, first the newly developed X080 Opsin MLPA was performed on ten male and ten female samples, of whom color vision status was unknown. For all probes fluorescent signals were detected at the expected fragment length and the ratios of the control probes between all samples were as expected between 0.8 and 1.2. Subsequently, the copy number of the LCR and *OPN1LW/OPN1MW* genes was determined in all samples, by taking four male samples with comparable low fluorescent signals for all targets in the opsin locus as a reference, with the assumption that these had one LCR, one *OPN1LW*, and one *OPN1MW* gene copy. Long-read sequence analysis of the *OPN1LW/OPN1MW* gene cluster performed in the four male control samples confirmed that the targets of the MLPA probes were correctly identified, moreover no heterozygous variants were present in these four male samples, which is also indicative that these males have one *OPN1LW* and one *OPN1MW* gene copy. Next, copy numbers were determined in the residual samples using these four male samples as a reference. In the remaining six male samples one LCR and three or four *OPN1LW*/*OPN1MW* gene copies were detected and in the ten female samples two LCR and four to eight *OPN1LW*/*OPN1MW* gene copies were detected.

To confirm that the *OPN1LW*/*OPN1MW* copy numbers can correctly be determined by MLPA, optical genome mapping (OGM) was performed as a secondary independent genetic technique. OGM and MLPA were performed on a ‘genome in a bottle’ sample (NA12878)^28^ and four additional control samples. Data analysis was performed in a blinded way, and results were compared afterwards. *OPN1LW/OPN1MW* copy numbers of one, two, three, five, and six were detected, respectively, and all results were concordant between MLPA and OGM (Supplementary Fig. 2). These results confirm that the MLPA can correctly determine copy numbers of *OPN1LW* and *OPN1MW*.

### Copy number analysis in clinical samples

Secondly, the Opsin MLPA was performed on 50 clinical samples (43 male and 7 female samples). In two out of 43 male clinical samples, both probe signals of the LCR were absent indicating a deletion of the LCR. Of the remaining 41 male samples, ten had a single *OPN1LW*/*OPN1MW* gene copy, 16 had two, and 15 had three or more *OPN1LW*/*OPN1MW* gene copies (Supplementary Table 1). The seven female samples had two LCR and four to six *OPN1LW*/*OPN1MW* gene copies (Supplementary Table 2).

### Specificity of the long-read sequencing amplicons

**Fig. 2:**
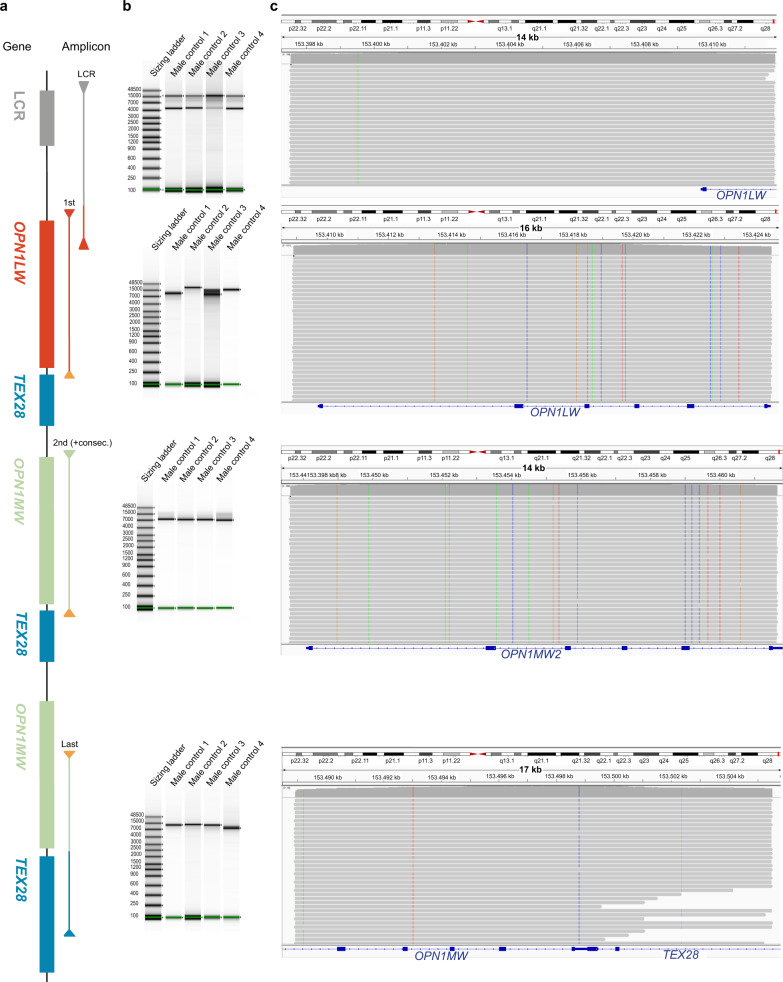
Gel electrophoresis and sequencing data of all four amplicons. **a** Schematic overview of the *OPN1LW/OPN1MW* gene cluster. The location of the four amplicons designed for the *OPN1LW/OPN1MW* gene cluster are depicted as bars beginning and ending with an arrow. **b** Gel electrophoresis results of four male control samples for each of the four amplicons and a sizing ladder (in base pairs). For each amplicon PCR and sequencing was done in parallel for all samples. Moreover for each amplicon PCR products of all samples were run on a single DNA ScreenTape Analysis. **c** Long-read sequencing results of one of the previously mentioned control samples shown in IGV for each of the amplicons. On the top the chromosomal location is depicted and on the bottom the specific gene(s) located at this region is/are shown.

Since causal nucleotide variants have also been described in the opsin locus, four LR amplicons were designed to cover the *OPN1LW*/*OPN1MW* gene cluster (Fig. 1a). The specificity of these amplicons was determined on four male control samples with one *OPN1LW* and one *OPN1MW*. All four LR-PCR reactions yielded amplicons of the expected length (Fig. 2). Moreover, the sequencing reads of the amplicons for the first and second (and consecutive) opsin gene aligned only to the opsin locus when mapped to the entire genome (Supplementary Fig. 3). Although the amplicon for the last opsin gene yielded a single band, next to the alignment of most reads to the opsin locus, some reads also aligned to chromosome 1. The amplicon for the LCR showed an additional a-specific product of ~6 kb (Fig. 2), the reads of this product aligned to an intronic region of *IGF1R* at chromosome 15.

### Variant analysis in clinical samples

**Fig. 3:**
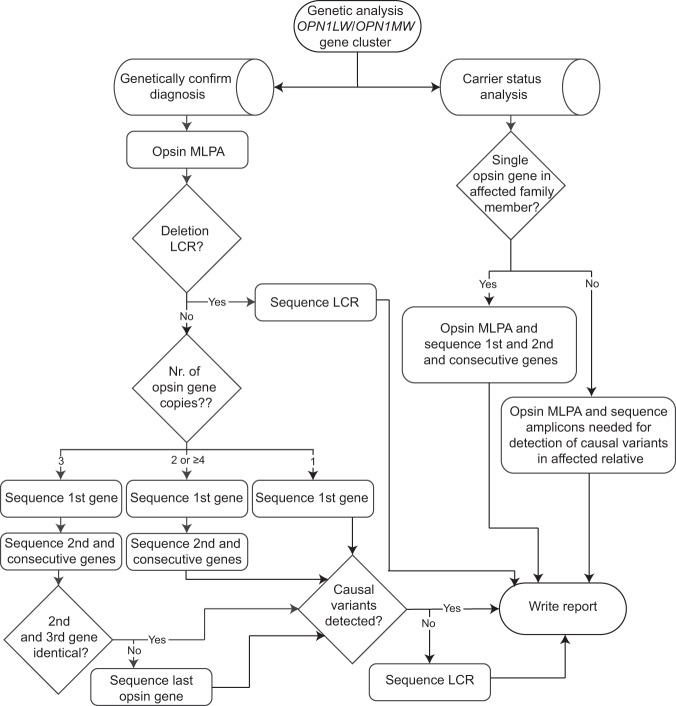
Flow diagram to determine which genetic tests need to be performed for a clinical sample. The following sequencing strategy was developed for samples in which the clinical question was to genetically confirm the clinical diagnosis: (1) If a deletion of the LCR was detected by MLPA the LCR amplicon is sequenced. (2) In samples with a LCR (determined by MLPA) the first gene amplicon is sequenced. (3) In samples with 2 or more genes (determined by MLPA) the second (and consecutive) gene(s) amplicon is sequenced. (4) In samples with three opsin gene copies in the cluster (determined by MLPA) and in which the last two gene copies are not identical (determined by sequencing of second (and consecutive) gene(s) amplicon) the last gene amplicon is sequenced. (5) In samples in which no causal variants are detected (determined by MLPA and sequencing of the first gene, the second (and consecutive) gene(s) and/or last gene amplicons) the LCR amplicon is sequenced. In samples in which the clinical question was carrier status analysis the sequencing strategy depends on the genetic results of the affected relative. In all samples MLPA needs to be performed to determine the number of opsin gene copies. When a single (hybrid) gene is detected in the affected family member, the first gene amplicon and second (and consecutive) gene(s) amplicon need to be sequenced as single (hybrid) genes frequently occur de novo. In the other cases the same amplicons need to be sequenced that were needed to detect causal variants in the affected family member.

A sequencing strategy to determine which sequence reactions per clinical sample is needed is explained in detail in Fig. 3, and the 50 clinical samples were sequenced accordingly. Details on mapping statistics of amplicons sequenced for each of the clinical samples are stated in Supplementary Table 3.

In two samples (USN04466 and USN15024) the LCR amplicon was performed as the MLPA determined the absence of the LCR. No PCR product was amplified, most likely the deletions in these samples also include the genomic sequence to which the forward and/or reverse primer of the LCR amplicon bind.

In 48 samples the first gene amplicon was sequenced and in all 48 samples the first gene in the cluster was a (hybrid) *OPN1LW* (Supplementary Tables 1 and 2). In 39 samples variants were detected in the (hybrid) *OPN1LW*, including a variant of unknown significance (VUS) in the promoter region (c.-33_-13delinsATCAC), protein truncating variants (c.802_804delinsG p.(Arg268fs) and c.852C > A p.(Tyr284*)), the pathogenic c.607T > C p.(Cys203Arg) variant, missense VUS (c.347C > G p.(Ser116Cys) and c. 1013G > T p.(Gly338Val)), pathogenic combinations of variants LIAVA, LIVVA, LVAVA, MIAVA and MVAVA and the MVVVA combination of unknown significance (Supplementary Tables 1 and 2).

Sequencing of the second (and consecutive) gene(s) amplicon was performed in 38 clinical samples. As one to four genes were sequenced simultaneously, the detected variant percentages ranged from ~25% to 100%. Using the variant percentage, it was deduced whether one, multiple or all gene copies had the variant(s). In all 38 samples the second (and consecutive) gene(s) in the cluster was/were (a) (hybrid) *OPN1MW* (Supplementary Tables 1 and 2). In 29 samples nucleotide variants were detected, including the pathogenic c.607T > C p.(Cys203Arg) variant, the missense VUS c.38G > A p.(Arg13His), and c.659T > C p.(Met220Thr), pathogenic combinations of variants LVAVA, MIAVA and MVAVA and the MVVVA combination of unknown significance (Supplementary Tables 1 and 2).

Sequencing of the last gene amplicon was performed in five male samples with three opsin genes, in which the latter two genes were not identical. Because the reverse primer of this amplicon is located outside the duplicated region and because in all samples only one of the two earlier detected (hybrid) *OPN1MW* genes was sequenced (Supplementary Table 1), we assume that the amplicon is specific for the last opsin gene of the cluster. In two samples the last gene was the *OPN1MW* gene copy without the pathogenic variant, in one sample a hybrid *OPN1MW*, and in the last two samples an *OPN1MW* with the MVAVA combination (Supplementary Table 1).

In two samples (USN01345 and USN20083) in which no causal variants were detected and in two samples (USN15983 and USN03197) where it was unclear whether casual variants were detected, sequencing of the LCR was performed to exclude a small deletion of the LCR that could have been missed by MLPA. No deletions or rare variants were detected in the LCR of these samples.

### Define *OPN1LW*/*OPN1MW* gene cluster in clinical samples

**Fig. 4:**
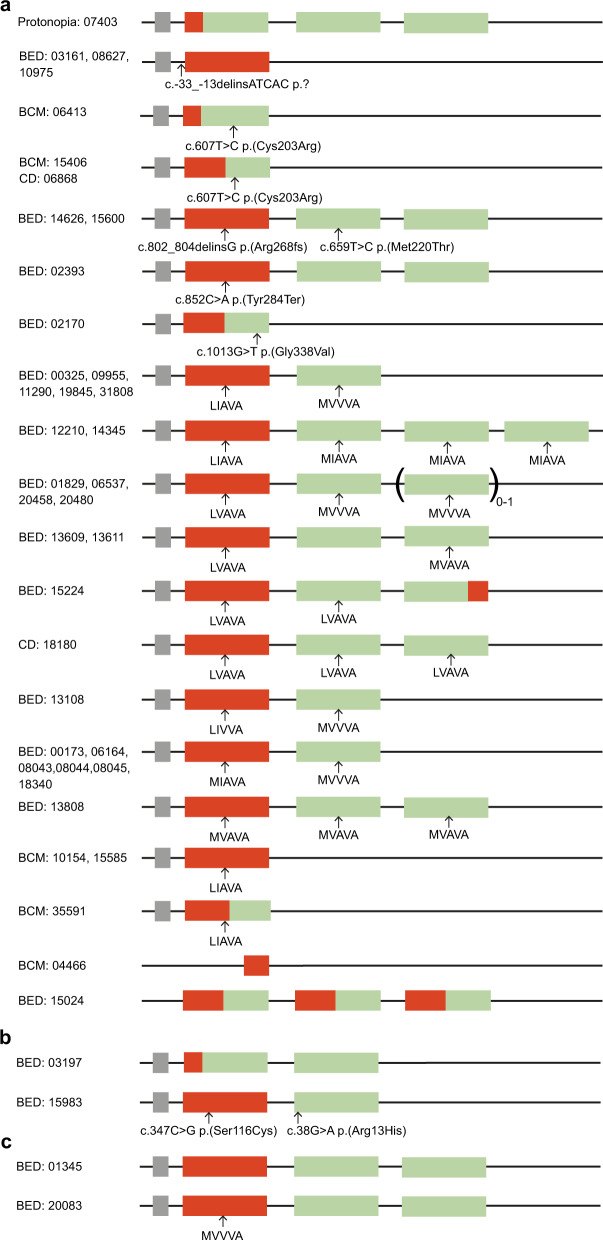
Schematic overview of the alleles detected in the 43 clinical samples in whom genetic analysis of the *OPN1LW*/*OPN1MW* gene cluster was performed to determine the genetic cause of their visual impairment. **a** Causative alleles detected in 39 patients with protanopia, BED, BCM, and/or cone dystrophy. **b** Alleles detected in two samples of which it is unknown whether they are causal. **c** Two samples in which detected alleles are not causal for the inherited visual impairment of the patient.

The results of the MLPA and long-read sequencing were combined to determine the genetic composition of the *OPN1LW*/*OPN1MW* gene cluster in the 50 clinical samples. In 43 samples the clinical question was to genetically confirm the clinical diagnosis of protanopia, BED, BCM or cone dystrophy. In 39 out of these 43 patient samples causal variants were detected (Fig. 4a), in one case causality remained uncertain due to the detection of a VUS in both *OPN1LW* and *OPN1MW* (Fig. 4b), in one case a genetic cause for protanopia was detected while the patient was referred to us with BED (Fig. 4b) and in two samples a benign *OPN1LW*/*OPN1MW* allele was identified (Fig. 4c).

**Fig. 5:**
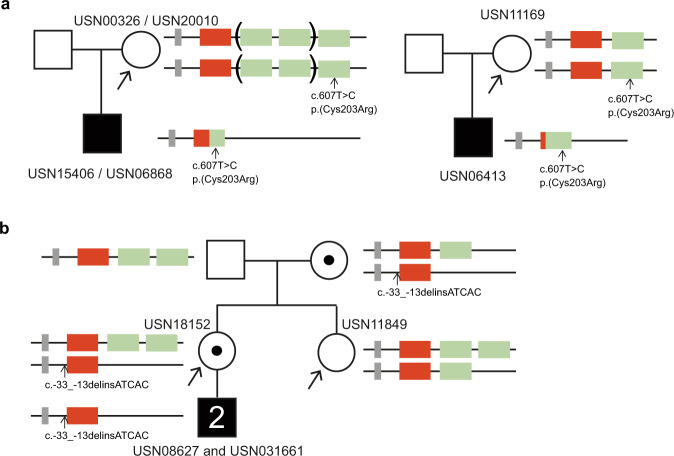
Schematic overview of the pedigree and detected alleles in four clinical samples and relatives in whom carrier status analysis of the *OPN1LW*/*OPN1MW* gene cluster was performed. The individuals in whom carrier status analyses was requested are depicted with the arrow. **a** In three independent cases, USN00326, USN11169, and USN20010, each with a son with BCM while there was no family history of BCM, the hybrid allele with the pathogenic c.607T > C variant detected in their sons arose due to a gene recombination event in the germline or early embryonic development. **b** Carrier status analysis in two siblings USN18152 and USN11849. USN18152 is the mother of two affected children and carried a VUS also detected in her sons, while her sister USN11849 did not have the VUS, indicating that USN18152 was a carrier and USN11849 not. As with the current method, we can not determine the amount of opsin gene copies on each of the alleles in females, analysis of their parents (grandparents of the affected boys) was needed, to definitely determine carrier status. When the parental samples were tested it was determined that USN18152 was a carrier and USN11849 not.

In seven samples the clinical question was to determine carrier status. Interestingly, in three samples that were tested for carrier status for BCM, because they each had a son with BCM without any family history of BCM, a gene recombination event had taken place in the germline or early embryonic development giving rise to a hybrid allele with the pathogenic c.607T > C p.(Cys203Arg) variant in their affected son (Fig. 5a, Supplementary Table 2). Furthermore, also in USN19477 a yet undefined genomic event had taken place in the germline or early embryonic development as in this sample the LIAVA sequence present in the single opsin gene in her son was not detected in any one of her opsin gene copies (Supplementary Table 2). USN18152 with two affected children was a carrier of BED, while her sister inherited a benign allele from their mother (Fig. 5b, Supplementary Table 2). The remaining sample (USN09034) probably inherited a benign allele from her mother who was an obligate carrier (Supplementary Table 2).

## Discussion

The developed assay for the highly complex genetic *OPN1LW/OPN1MW* cluster is a diagnostic genetic test that can detect a wide range of pathogenic variants in the cluster due to the combination of copy number analysis and long-read sequencing. The analysis is straightforward as MLPA is a fast and efficient method for copy number analysis and the long-read sequencing approach makes it possible to determine the genetic composition of the entire *OPN1LW* or *OPN1MW* gene in single sequencing reads. The developed assay improves diagnostic care of patients with cone dysfunction and may help clinicians in the identification of future patients in which genetic analysis of the *OPN1LW/OPN1MW* gene cluster is required.

Copy number analysis of the *OPN1LW/OPN1MW* gene cluster was performed using a newly developed MLPA test. Control samples all had copy numbers in the range described in persons with normal color vision^18^, while in clinical samples aberrant *OPN1LW/OPN1MW* gene copy numbers were detected. Next, long-read sequencing reactions were developed for variant analysis of the *OPN1LW/OPN1MW* cluster. Mapping of sequencing reads was performed on an artificial genomic reference sequence consisting of one *OPN1LW* and one *OPN1MW*, as the genomic reference sequences of GRCh37 and GRCh38 where both not suitable for mapping of sequencing reads. Both GRCh37 and GRCh38 contain multiple *OPN1MW* gene copies (GRCh37: 2 *OPN1MW* gene copies, GRCh38: 3 *OPN1MW* gene copies) and sequencing reads from the second (and consecutive) gene(s) amplicon are randomly assigned to one of the *OPN1MW* gene copies, giving rise to incorrect variant percentages.

As only the first two opsin genes downstream of the LCR are translated into protein^4^, the genetic composition of only the first two genes in the cluster is clinically relevant. Even with the long-range approach that was used, spanning amplicons over 16 kb, no amplicon specific for the second gene in the cluster could be developed. However, most clinical samples only have two opsin genes in their cluster, and in those samples the second (and consecutive) gene(s) amplicon will only render results of the second gene as there are no consecutive genes (Fig. 1b). An additional LR-PCR was developed to deduce the genetic make-up of the second gene in samples which have three opsin genes (Fig. 1c). Although the developed diagnostic assay can’t determine the exact composition of the cluster when four or more opsin gene copies are present and the genetic composition of the second and consecutive gene copies are not identical (Fig. 1d), the assay was able to determine the composition of the first two genes in the *OPN1LW/OPN1MW* gene cluster in all 43 tested male clinical samples, indicating that in the vast majority of patients this assay is sufficient for the analysis of casual haplotypes in the *OPN1LW/OPN1MW* gene cluster.

As no commercially available reference samples or quality control schemes are available specifically for the *OPN1LW*/*OPN1MW* gene cluster, the performance of our newly developed genetic assay was validated in two steps. First, the performance of MLPA was tested with a second independent genetic technique, OGM. Five samples were analyzed by both MLPA and OGM and identical *OPN1LW*/*OPN1MW* copy numbers were detected in all samples (Supplementary Fig. 2). These results proof that the new MLPA can correctly determine *OPN1LW*/*OPN1MW* copy numbers. Subsequently, the combination of MLPA and long-read sequencing was performed on a set of 50 clinical samples. In the 43 patients in whom reason for referral was genetic confirmation of the diagnosis a wide range of causal haplotypes were detected: deletions encompassing the LCR, aberrant gene array patterns, the presence of hybrid genes with or without pathogenic variants, single variants in one or more of the gene copies (including 7 variants that have not been published before), and the presence of a combination of variants in one or more gene copies (Fig. 4). In 39 of the 43 patients the clinical diagnosis was genetically confirmed, in two patients it was unclear whether the detected variants in the cluster were causal and in the remaining two patients no clinical relevant variants were detected. In these last two samples, most likely the genetic cause of the visual impairment is outside the *OPN1LW*/*OPN1MW* gene cluster. Indeed, in USN01345 exome sequencing was performed and two VUS’s were detected in the *ZNF644* gene (Chr1(GRCh37):g.91404863C > G NM_201269.2:c.2048G > C (p.(Arg683Thr)) and Chr1(GRCh37):g.91382477T > C NM_201269.2:c.3862A > G (p.(Ile1288Val)), respectively). We therefore can conclude that the developed genetic test is able to detect causal nucleotide, structural and copy number variants in the *OPN1LW*/*OPN1MW* gene cluster.

Genetic analysis of the *OPN1LW*/*OPN1MW* gene cluster for carrier status is even more complex as women have two alleles with an *OPN1LW*/*OPN1MW* gene cluster and with our developed genetic assay we are unable to determine how many opsin gene copies are on each allele. This is underscored in sisters USN18152 and USN11849, in whom additional samples were needed to be analyzed to determine that USN18152 is a carrier due to presence of an allele with a single *OPN1LW* and no *OPN1MW*, while USN11849 is not (Fig. 5b). It is of clinical importance to perform carrier status analysis of the *OPN1LW*/*OPN1MW* gene cluster to determine recurrence risk, especially in families with no family history of visual impairment, as shown in four cases in whom the pathogenic allele arose *de novo*.

As shown in Supplementary Fig. 2, the next generation cytogenetics technology OGM^29,30^ is capable to determine total *OPN1LM/OPN1MW* gene copy numbers. As this technology relies, however, on ultra-long DNA molecules (N50 > 150 kb), conventional isolated DNA cannot be used, and fresh material (either blood or cell lines) is needed. For the 50 samples mentioned in this paper, this fresh material was not available. If, in future, diagnostics for the *OPN1LW/OPN1MW* gene cluster can be arranged such that fresh material is available OGM could be an alternative for copy number and structural variant analysis. In addition, also long-read WGS could be an interesting alternative. At the moment however, the latter one provides too low coverage at too high cost. Moreover, for analysis of the *OPN1LW/OPN1MW* gene cluster it requires a reference-free de novo assembly, making analysis still not trivial.

The detection rate of causal variants in patients with inherited retinal dystrophies (IRDs) with a predominant cone involvement is low compared to patients with IRDs with a predominant rod involvement^31^. This could be due to the fact that short-read NGS is the principal diagnostic test used for patients with a visual impairment, while *OPN1LW* and *OPN1MW* have so called “NGS dead zones” genomic regions that can not be analyzed with short-read NGS^26^. It would be interesting to test IRD patients with a predominant cone involvement who have a negative NGS result with an adequate genetic test for the *OPN1LW/OPN1MW* gene cluster, to determine whether causal variants in the *OPN1LW/OPN1MW* gene cluster are underestimated in IRD patients. Next to *OPN1LW* and *OPN1MW*, another 617 genes of which 71 are medically relevant for various disease phenotypes have such NGS dead zones in their coding regions^26^. Clinicians should be aware that when short-read NGS is utilized the analysis may not be adequate for all clinically relevant genes.

## Methods

### DNA samples from clinical archive

A diagnostic laboratory can use (de-identified) archived clinical samples to validate and implement novel diagnostic assays. The derived clinically relevant variants can be shared, but in absence of explicit data-sharing consent at individual patient level, FASTQ, BAM and VCFs cannot be disclosed. These methods are also in accordance with relevant guidelines and regulations and approved by the institutional review board of the Radboud University Medical Center (2020-7142) and the Declaration of Helsinki.

All clinical samples (*n* = 50) were received between 2015 and 2020 by the Department of Human Genetics of the Radboud University Medical Center. In 43 male samples the genetic test was requested to genetically confirm the diagnosis of protanopia (*n* = 1), BED (*n* = 32), BCM (*n* = 8) or cone dystrophy (*n* = 2). The type of visual impairment was diagnosed by expert ophthalmologists based on appropriate clinical examinations. In seven female samples the genetic test was requested for carrier status analysis, because of multiple affected children (*n* = 1), affected child without a positive family history (*n* = 4) or affected or carrier sibling (*n* = 2). Genomic DNA isolation of peripheral blood was performed in an automated manner as described previously^32^. In short, genomic DNA isolation was performed automatically using a HamiltonMicrolab STARautoload system with an integrated Chemagen MSM I separation module (Hamilton Robotics GmbH). DNA isolation was performed using the Chemagic DNA blood kit special (PerkinElmer) according to the manufacturer’s instructions. For the purpose of this study, the samples were anonymized and coded with a unique study number (USN).

### MLPA

A MLPA test specific for the *OPN1LW*/*OPN1MW* gene cluster was developed together with MRC Holland. The SALSA MLPA Probemix X080 Opsin consists of 9 control probes and 16 probes targeting the *OPN1LW*/*OPN1MW* gene cluster, all located on the X-chromosome. Two probes target the LCR. Six probes target *OPN1LW* (NM_020061.5): c.-77A in exon 1, c.300A in exon 2, c.457C in exon 3, c.706A in exon 4, and c.830 A and c.926A in exon 5. Six probes target *OPN1MW* (NM_000153.2): c.-62A in exon 1, c.300G in exon 2, c.457A in exon 3, c.706 G in exon 4, and c.830T and c.926T in exon 5. Two probes target both *OPN1LW* and *OPN1MW*: c.607T in exon 4, and c.1083A in exon 6 (Fig. 1a). Probe sequences are stated in Supplementary Table 4. For the probes targeting c.457 and c.706 of both *OPN1LW* and *OPN1MW* single nucleotide variants have been described within 10 nucleotides of the target. Accordingly, there is a possibility that when such a variant is present this will affect the performance of the probe.

The MLPA reaction was performed according to the manufacturer’s protocol (MRC Holland) on a thermocycler (Geneamp PCR system 9700 (ThermoFisher) or Veriti 96 well thermal cycler (Applied Biosystems)). In short, 50–100 ng DNA was denatured and mixed with 1.5 µl SALSA MLPA buffer and 1.5 µl probe mix. After 16 to 20 hours of hybridization at 60 °C, 3 µl SALSA ligase buffer A, 3 µl SALSA ligase buffer B, 1 µl SALSA ligase-65 and 25 µl H_2_O was added and incubated for 15 min at 54 °C. Finally, a polymerase chain reaction (PCR) was performed by adding 2 µl SALSA PCR primer mix, 0.5 µl SALSA polymerase, and 7.5 µl H_2_O. The PCR protocol was: 35 cycles of 30 seconds at 95 °C, 30 s at 60 °C and 1 min at 72 °C, followed by a final elongation step of 20 min at 72 °C. A mixture of 1 µl MLPA sample and 8.8 µl formamide (Hi-Di, Applied Biosystems) and 0.2 µl size standard (GeneScan 500-Liz, Applied Biosystems) was analyzed on a fragment analyzer (Model 3130, Applied Biosystems). Genemarker (V2.6.7, Softgenetics) was used for data analysis.

### Long-range PCR

Four long-range PCRs (LR-PCRs) were designed to encompass the *OPN1LW*/*OPN1MW* gene cluster (Fig. 1a). One amplicon of 14,374 bp was designed specific for the LCR region (primers 099-692 GCAAAGGCTCTTCCTTTGTG and 099-693 AGGTGAAGGCAGGGTAAGGT). One amplicon of 15,634 bp specifically spans the first opsin gene copy (most often a (hybrid) *OPN1LW*) in the cluster (primers 044-982 GAGGCGAGGCTACGGAGT and 044-984 GCAGTGAAAGCCTCTGTGACT). One amplicon of 14,034 bp spans the second and if present consecutive opsin gene copies (most often (hybrid) *OPN1MW*) in the cluster (primers 080-273 AGCTGGGAGTACAGGTATTTG and 044-984). Lastly, one amplicon of 16,589 bp was designed to identify part of the last opsin gene (most often a (hybrid) *OPN1MW*) in the cluster (primers 099-820 AGGTGTAGAGCCCTAGCAAAC and 099-821 TCTCATTCATAAATTGCTGGTA).

PCR was as follows: 100 ng DNA, 12.5 µl LongAmp Hot Start Taq 2x Master Mix (Bioke), 2 µl 10 µM forward and reverse primer, 10.5 µl H_2_O. The PCR protocol for LCR, first gene, and second and consecutive genes was: 94 °C for 1 min, followed by 30 cycles of 30 s at 94 °C and 14 min at 65 °C, followed by a final elongation step of 10 min at 65 °C. The PCR protocol for the last gene was: 94 °C for 1 min, followed by 30 cycles of 30 s at 94 °C, 1 min 56 °C, and 15 min at 65 °C, followed by a final elongation step of 10 min at 65 °C. All amplicons were checked on agarose gel or DNA ScreenTape Analysis (TapeStation, Agilent).

### Library preparation

LR amplicons were purified by AMPure PB beads (Pacific Biosciences), using a bead ratio of 1.5x. Library preparation was done according to protocol ‘Procedure and Checklist—Preparing SMRTbell Libraries using PacBio Barcoded Adapters for Multiplex SMRT Sequencing’ (Pacific Biosciences, Part Number 100-538-700-02). In brief, sample concentrations were measured with Qubit 3.0 (ThermoFisher Scientific). Equimolar amounts of amplicons, adding up to a total input of 200 ng to 1 ug DNA, were used to perform one-step end repair and adapter ligation (using barcoded hairpin adapters), followed by equimolar pooling. The pool was purified with AMPure PB beads, followed by DNA damage repair, exonuclease digestion, and an additional two rounds of AMPure PB beads purification.

### Generation of polymerase-bound SMRTbell complexes and SMRT sequencing

Generation of polymerase-bound SMRTbell complexes was performed using the Sample Setup option in SMRTLink (Pacific Biosciences). In brief, sequencing primers were conditioned and annealed to the SMRTbell library, followed by dilution and binding of the sequencing polymerase. The polymerase bound complex was purified using AMPure PB beads, and concentration was measured via Qubit. An internal control sample was diluted and added to the polymerase-bound complex, together with DTT, Sequel additive, and complex dilution buffer.

Sequencing was performed using the Run Design option in SMRTLink. Libraries were loaded using diffusion loading with an on plate concentration of 4.5 pM. All runs were sequenced using a movie time of 20 h per SMRTcell and included pre-extension. Sequencing was performed on a Sequel I system (Pacific Biosciences) with ICS version 6.0. The amplicons of the *OPN1LW/OPN1MW* gene cluster were sequenced together with other types of long-read amplicons. Sequencing runs were performed twice a week, irrespective of the number of samples available, with a maximum of 24 different barcodes per sequencing run, a barcode can contain more than one amplicon. Accordingly, coverage varies between the different samples. The low number of barcodes however guarantees sufficient coverage for each analysis. The minimum coverage required for data analysis was 20x.

### Data analysis and variant calling

Following sequencing, raw data was analyzed using CCS mapping in SMRTLink. Analysis was performed using default settings with minor modifications: only reads longer than 10 kb were mapped and for CCSmapping the selected reference was an adapted GRCh37 with only one copy of *OPN1MW*. The additional setting ‘PlaceGapConsistently’ was added to the algorithm options in order to allow left alignment, and ‘consolidate bam’ was set to ‘ON’ (default: OFF). Generated bam files were uploaded into SeqNext (JSI Medical systems) and variant calling was performed using default settings on *OPN1LW* (NM_020061.6) and *OPN1MW* (NM_000513.2).

### Optical genome mapping

Optical genome mapping of control samples S3.1 to S3.4 was performed as described previously^29^, with minor modifications. In brief, ultra-high molecular weight DNA was isolated from 650 ul peripheral blood (EDTA) using the SP Blood and Cell Culture DNA Isolation Kit according to manufacturers’ instructions (Bionano Genomics). Per sample, 750 ng of DNA was labeled with the DLS (Direct Label and Stain) DNA Labeling Kit (Bionano Genomics), and labeled DNA was imaged on the Saphyr System (Bionano Genomics) using ICS version 5.2. The annotated de novo assembly pipeline was executed with Bionano Solve software 3.6.1. Data of genome in a bottle sample NA12878^28^ is provided by courtesy of Bionano Genomics. Quality statistics of the samples are stated in Supplementary Table 5.

### Reporting summary

Further information on research design is available in the Nature Research Reporting Summary linked to this article.

### Supplementary information

Reporting summary

Supplementary information

Supplementary tables

## Data Availability

The individual-level sequencing and optical genome mapping data are available behind the Radboud University Medical Center firewall. In absence of explicit data-sharing consent at individual patient level, FASTQ, BAM, and VCFs cannot be disclosed. These data are, however, available for review at or via a secured connection with the department of Human Genetics of the Radboud University Medical Center. Data is available upon reasonable request and after a data usage agreement through corresponding author L.H.-W. All sequencing variants that were considered to be potentially pathogenic are available in Supplementary Tables 1 and 2.
