# Supplementary material for: Diagnostic analysis of the highly complex OPN1LW/OPN1MW gene cluster using long-read sequencing and MLPA
Source: NPJ Genom Med. 2022 Nov 9;7:65. doi: 10.1038/s41525-022-00334-9 (PMC9646815; doi:10.1038/s41525-022-00334-9)
Supplement: Supplementary file 1 — Reporting summary [file 41525_2022_334_MOESM1_ESM.pdf]

nature portfolio

NPJGENMED-01432

Corresponding author(s): L.Haer-Wigman

Last updated by author(s): Aug 30, 2022

Reporting Summary

Nature Portfolio wishes to improve the reproducibility of the work that we publish. This form provides structure for consistency and transparency in reporting. For further information on Nature Portfolio policies, see our Editorial Policies and the Editorial Policy Checklist.

Statistics

For all statistical analyses, confirm that the following items are present in the figure legend, table legend, main text, or Methods section.

n/a	Confirmed	
☐	☒	The exact sample size (*n*) for each experimental group/condition, given as a discrete number and unit of measurement
☐	☒	A statement on whether measurements were taken from distinct samples or whether the same sample was measured repeatedly
☒	☐	The statistical test(s) used AND whether they are one- or two-sided<br>*Only common tests should be described solely by name; describe more complex techniques in the Methods section.*
☒	☐	A description of all covariates tested
☒	☐	A description of any assumptions or corrections, such as tests of normality and adjustment for multiple comparisons
☒	☐	A full description of the statistical parameters including central tendency (e.g. means) or other basic estimates (e.g. regression coefficient) AND variation (e.g. standard deviation) or associated estimates of uncertainty (e.g. confidence intervals)
☒	☐	For null hypothesis testing, the test statistic (e.g. *F*, *t*, *r*) with confidence intervals, effect sizes, degrees of freedom and *P* value noted<br>*Give P values as exact values whenever suitable.*
☒	☐	For Bayesian analysis, information on the choice of priors and Markov chain Monte Carlo settings
☒	☐	For hierarchical and complex designs, identification of the appropriate level for tests and full reporting of outcomes
☒	☐	Estimates of effect sizes (e.g. Cohen's *d*, Pearson's *r*), indicating how they were calculated

Our web collection on statistics for biologists contains articles on many of the points above.

Software and code

Policy information about availability of computer code

Data collection	MLPA samples were analyzed on a fragment analyzer (Model 3130, Applied Biosystems). Genemarker (V2.6.7, Softgenetics). All amplicons were checked on agarose gel or DNA ScreenTape Analysis (TapeStation, Agilent). Long-read sequencing was performed using the Run Design option in SMRTLink. and was performed on a Sequel I system (Pacific Biosciences) with ICS version 6.0. Optical genome mapping was performed on the Saphyr System (Bionano Genomics) using ICS version 5.2.
Data analysis	Genemarker (V2.6.7, Softgenetics) was used for data analysis of MLPA results. Long-read sequencning raw data was analyzed using CCS mapping in SMRTLink. Generated bam files were uploaded into SeqNext (JSI Medical systems) and variant calling was performed using default settings on OPN1LW (NM_020061.6) and OPN1MW (NM_000513.2). The annotated de novo assembly pipeline was executed with Bionano Solve software 3.6.1. was performed on the optical genome mapping results.

For manuscripts utilizing custom algorithms or software that are central to the research but not yet described in published literature, software must be made available to editors and reviewers. We strongly encourage code deposition in a community repository (e.g. GitHub). See the Nature Portfolio guidelines for submitting code & software for further information.

Data

Policy information about availability of data

All manuscripts must include a data availability statement. This statement should provide the following information, where applicable:

- Accession codes, unique identifiers, or web links for publicly available datasets
- A description of any restrictions on data availability
- For clinical datasets or third party data, please ensure that the statement adheres to our policy

The individual-level sequencing and optical genome mapping data are available behind the Radboudumc firewall and are prohibited for external sharing due to patient privacy restrictions. These data are, however, available for review at the department of Human Genetics of the Radboudumc. Other data are available upon request and can be obtained from corresponding author L.H-W. upon reasonable request. All sequencing variants that were considered to be potentially pathogenic are available in the Supplementary Tables 1 and 2.

Human research participants

Policy information about studies involving human research participants and Sex and Gender in Research.

Reporting on sex and gender	As the OPN1LW/OPN1MW gene cluster is located on the X-chromosome. the sex of each indivudal is stated. Moreover the clincial question differs for male (confirmation of clinical diagnosis) and female (carrier status analysis) individuals, which is stated in the manuscript
Population characteristics	Patient with a clinical question regarding the OPN1LW/OPN1MW gene cluster were included in this study.
Recruitment	All clinical samples (n=50) were received between 2015 and 2020 by the Department of Human Genetics of the Radboudumc. In 43 male samples the genetic test was requested to genetically confirm the diagnosis of protanopia (n=1), BED (n=32), BCM (n=8) or cone dystrophy (n=2). The type of visual impairment was diagnosed by expert ophthalmologists based on appropriate clinical examinations. In seven female samples the genetic test was requested for carrier status analysis, because of multiple affected children (n=1), affected child without a positive family history (n=4) or affected or carrier sibling (n=2).
Ethics oversight	Radboud University Medical Cente

Note that full information on the approval of the study protocol must also be provided in the manuscript.

Field-specific reporting

Please select the one below that is the best fit for your research. If you are not sure, read the appropriate sections before making your selection.

☒ Life sciences ☐ Behavioural & social sciences ☐ Ecological, evolutionary & environmental sciences

For a reference copy of the document with all sections, see nature.com/documents/nr-reporting-summary-flat.pdf

Life sciences study design

All studies must disclose on these points even when the disclosure is negative.

Sample size	50 patien and 25 control samples were included
Data exclusions	no data was excluded
Replication	No replication was performed
Randomization	No randomization was performed
Blinding	No blinding was performed

Reporting for specific materials, systems and methods

We require information from authors about some types of materials, experimental systems and methods used in many studies. Here, indicate whether each material, system or method listed is relevant to your study. If you are not sure if a list item applies to your research, read the appropriate section before selecting a response.

Materials & experimental systems

n/a	Involved in the study
☒	☐ Antibodies
☒	☐ Eukaryotic cell lines
☒	☐ Palaeontology and archaeology
☒	☐ Animals and other organisms
☒	☐ Clinical data
☒	☐ Dual use research of concern

Methods

n/a	Involved in the study
☒	☐ ChIP-seq
☒	☐ Flow cytometry
☒	☐ MRI-based neuroimaging
