# Supplementary material for: Diagnostic analysis of the highly complex OPN1LW/OPN1MW gene cluster using long-read sequencing and MLPA
Source: NPJ Genom Med. 2022 Nov 9;7:65. doi: 10.1038/s41525-022-00334-9 (PMC9646815; doi:10.1038/s41525-022-00334-9)
Supplement: Supplementary file 2 — Supplementary information [file 41525_2022_334_MOESM2_ESM.pdf]

Supplementary Information Description

Supplementary Information includes 3 figures and 5 tables.

Supplementary Figure 1 Example of short read exome sequencing data in a male patient who has one hybrid *OPN1LW* and one *OPN1MW* with the pathogenic c.607T>C p.(Cys203Arg) variant

Supplementary Figure 2 Copy number analysis of the *OPN1LW/OPN1MW* cluster via MLPA and OGM

Supplementary Figure 3 Coverage across chromosomes thumbnails of the results of genome wide mapping of reads for each of the four amplicons

Supplementary Table 1 Genotyping results of patients affected with visual impairment with color deficiencies

Supplementary Table 2 Carrier analysis in females for the *OPN1LW/OPN1MW* gene cluster

Supplementary Table 3 Quality parameters of long-read sequencing of the amplicons

Supplementary Table 4 Probe sequences of the MLPA of the *OPN1LW/OPN1MW* gene cluster

Supplementary Table 5 Quality parameters of OGM

Supplementary Figure 1

a **b**

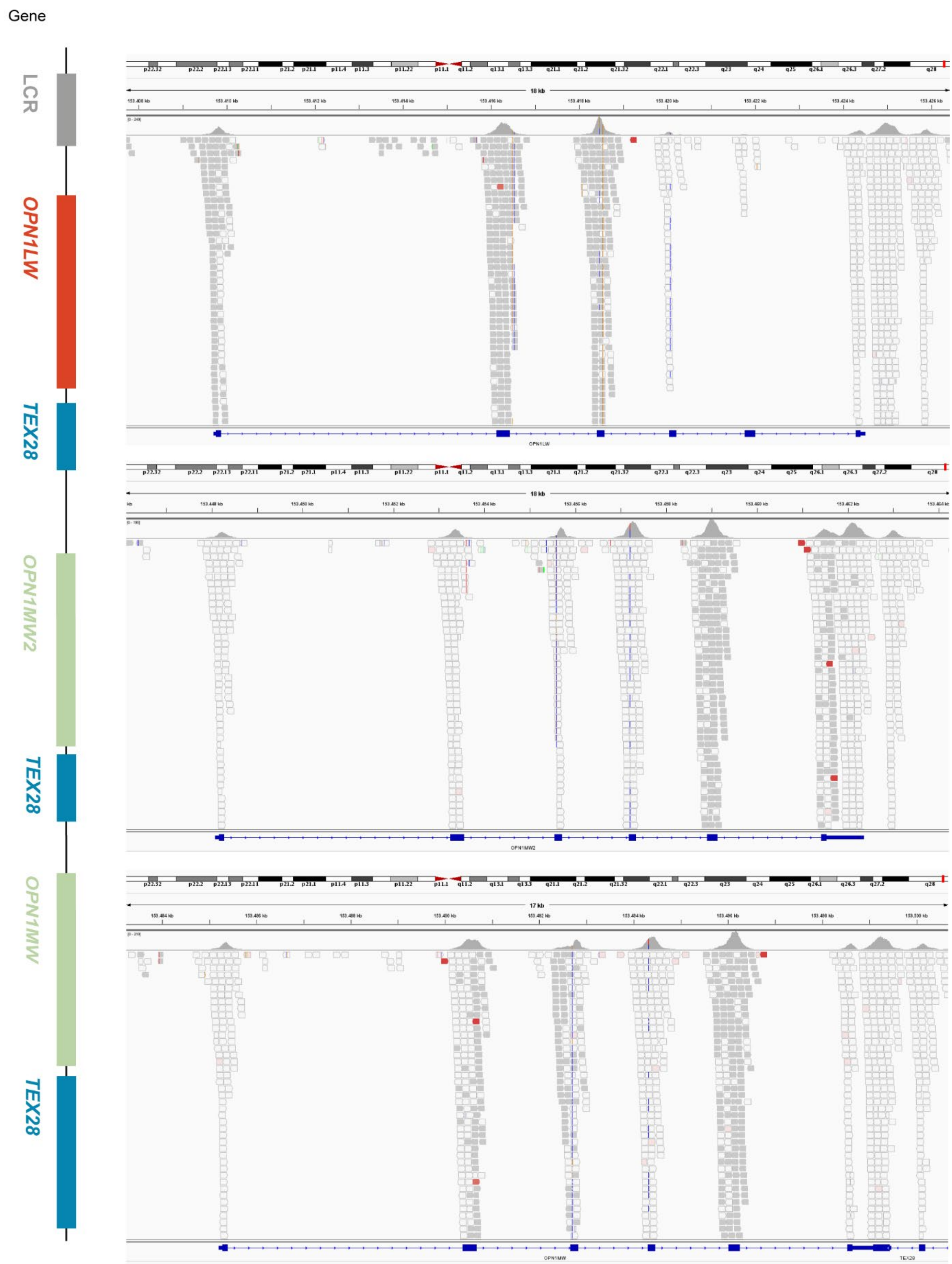

Supplementary Figure 1 Example of short read exome sequencing data in a male patient who has one hybrid *OPN1LW* and one *OPN1MW* with the pathogenic c.607T>C p.(Cys203Arg) variant. a Schematic overview of the *OPN1LW/OPN1MW* gene cluster. **b** Printscreens of short-read exome sequencing data aligned to *OPN1LW, OPN1MW2* and *OPN1MW* mapped on GRCh37. The example shows multiple reasons why short read sequencing data is inappropriate for analysis of the *OPN1LW/OPN1MW* gene cluster. 1) No reads are aligned to *OPN1LW* exon 4 and 5. CNV analysis on the exome data would incorrectly indicate the presence of a deletion of exon 4 and 5 of the *OPN1LW* gene. This patient has a *OPN1LW* hybrid gene copy where exon 4 and 5 have been interchanged from *OPN1MW* and therefore these reads are aligned to the *OPN1MW* reference. 2) (Most) reads of *OPN1LW* exon 6 and *OPN1MW2* and *OPN1MW* exon 1, 2, 3, 4 and 6 have a white color, indicating that the mapping quality of the reads is 0. When a read has a mapping quality of 0, variants in the read are not called by the variant detection software. The c.607T>C variant hemizygously present in the single *OPN1MW* gene of this patient was therefore not visible in the list of detected variants when the exome data was analyzed. 3) Although this patient has a single *OPN1MW* gene copy in his cluster, sequencing reads are aligned to both the *OPN1MW* and *OPN1MW2* gene of the reference sample, incorrectly indicating that this patient has two *OPN1MW* gene copies. As the *OPN1MW* and *OPN1MW2* genes are identical, reads are randomly assigned to either one of the genes. 5) Although the c.607T>C is not present in the list of variants detected in this sample, because the reads in which the variant is present have a read quality of 0, the c.607T>C variant is visible when the BAM/CRAM files are visually inspected. However, in the BAM/CRAM files the c.607T>C is incorrectly shown to be heterozygous present, because the reads of exon 4 of the hybrid *OPN1LW* gene copy are aligned to the *OPN1MW* gene.

Supplementary Figure 2

a

Control S3.1

	Probe Name	Bin Size	Height Ratio
1	**Ctrl_Xp11**	182.9	1.018
2	**Ctrl_Xp11_2**	215.0	0.958
3	**Ctrl_Xp22**	160.5	0.949
4	**Ctrl_Xq11**	335.9	1.007
5	**Ctrl_Xq21**	371.3	0.970
6	**Ctrl_Xq23**	127.1	0.995
7	**Ctrl_Xq26**	257.0	1.021
8	**Ctrl_Xq27**	301.2	0.988
9	LCR1	309.8	0.999
10	LCR2	354.9	0.979
11	LW/MW_ex4_c607T	174.2	0.468
12	LW/MW_ex6_1083A	153.8	0.528
13	LW_ex1_c.-77A	167.8	0.994
14	LW_ex2_c.300A	188.7	1.012
15	LW_ex3_c.457C	225.1	1.054
16	LW_ex4_c.706A	201.8	1.075
17	LW_ex5_c.830A	237.4	1.052
18	LW_ex5_c.926A	319.0	0.956
19	MW_ex1_c.-62A	147.6	0.000
20	MW_ex2_c.300G	195.4	0.000
21	MW_ex3_c.457A	231.4	0.000
22	MW_ex4_c.706G	208.0	0.000
23	MW_ex5_c.830T	243.5	0.000
24	MW_ex5_c.926T	325.1	0.000

Control S3.2

	Probe Name	Bin Size	Height Ratio
1	**Ctrl_Xp11**	182.9	1.024
2	**Ctrl_Xp11_2**	215.0	0.989
3	**Ctrl_Xp22**	160.5	0.984
4	**Ctrl_Xq11**	335.9	1.011
5	**Ctrl_Xq21**	371.3	0.993
6	**Ctrl_Xq23**	127.1	0.999
7	**Ctrl_Xq26**	257.0	1.048
8	**Ctrl_Xq27**	301.2	1.030
9	LCR1	309.8	1.009
10	LCR2	354.9	1.025
11	LW/MW_ex4_c607T	174.2	0.534
12	LW/MW_ex6_1083A	153.8	1.023
13	LW_ex1_c.-77A	167.8	0.912
14	LW_ex2_c.300A	188.7	1.019
15	LW_ex3_c.457C	225.1	1.922
16	LW_ex4_c.706A	201.8	0.000
17	LW_ex5_c.830A	237.4	0.000
18	LW_ex5_c.926A	319.0	0.000
19	MW_ex1_c.-62A	147.6	0.906
20	MW_ex2_c.300G	195.4	1.009
21	MW_ex3_c.457A	231.4	0.000
22	MW_ex4_c.706G	208.0	1.847
23	MW_ex5_c.830T	243.5	1.971
24	MW_ex5_c.926T	325.1	1.859

Control S3.3

	Probe Name	Bin Size	Height Ratio
1	**Ctrl_Xp11**	182.9	1.020
2	**Ctrl_Xp11_2**	215.0	0.929
3	**Ctrl_Xp22**	160.5	0.995
4	**Ctrl_Xq11**	335.9	0.975
5	**Ctrl_Xq21**	371.3	1.007
6	**Ctrl_Xq23**	127.1	0.982
7	**Ctrl_Xq26**	257.0	1.021
8	**Ctrl_Xq27**	301.2	0.939
9	LCR1	309.8	0.947
10	LCR2	354.9	1.070
11	LW/MW_ex4_c607T	174.2	1.406
12	LW/MW_ex6_1083A	153.8	1.411
13	LW_ex1_c.-77A	167.8	0.980
14	LW_ex2_c.300A	188.7	0.888
15	LW_ex3_c.457C	225.1	0.000
16	LW_ex4_c.706A	201.8	0.000
17	LW_ex5_c.830A	237.4	0.935
18	LW_ex5_c.926A	319.0	0.905
19	MW_ex1_c.-62A	147.6	1.858
20	MW_ex2_c.300G	195.4	1.620
21	MW_ex3_c.457A	231.4	1.600
22	MW_ex4_c.706G	208.0	2.014
23	MW_ex5_c.830T	243.5	1.853
24	MW_ex5_c.926T	325.1	1.705

b

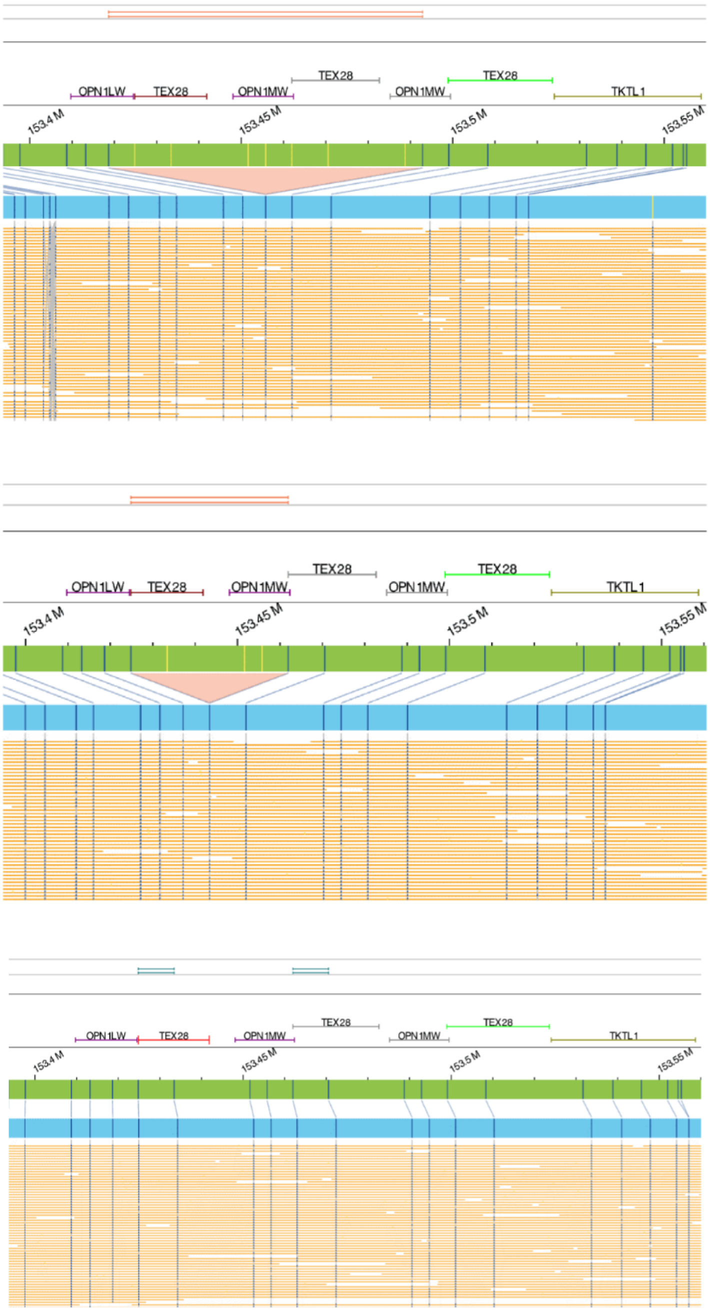

c

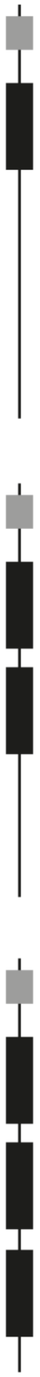

Supplementary Figure 2 (continued)

a

Control S3.4

	Probe Name	Bin Size	Height Ratio
1	**Ctrl_Xp11**	182.9	0.989
2	**Ctrl_Xp11_2**	215.0	1.020
3	**Ctrl_Xp22**	160.5	0.924
4	**Ctrl_Xq11**	335.9	1.066
5	**Ctrl_Xq21**	371.3	1.014
6	**Ctrl_Xq23**	127.1	0.976
7	**Ctrl_Xq26**	257.0	1.023
8	**Ctrl_Xq27**	301.2	0.978
9	LCR1	309.8	1.034
10	LCR2	354.9	1.000
11	LW/MW_ex4_c607T	174.2	0.992
12	LW/MW_ex6_1083A	153.8	1.225
13	LW_ex1_c.-77A	167.8	0.926
14	LW_ex2_c.300A	188.7	0.984
15	LW_ex3_c.457C	225.1	0.983
16	LW_ex4_c.706A	201.8	0.000
17	LW_ex5_c.830A	237.4	0.499
18	LW_ex5_c.926A	319.0	0.467
19	MW_ex1_c.-62A	147.6	1.280
20	MW_ex2_c.300G	195.4	1.433
21	MW_ex3_c.457A	231.4	1.445
22	MW_ex4_c.706G	208.0	2.018
23	MW_ex5_c.830T	243.5	1.878
24	MW_ex5_c.926T	325.1	1.826

GIAB sample NA12878

	Probe Name	Bin Size	Height Ratio
1	**Ctrl_Xp11**	182.9	1.044
2	**Ctrl_Xp11_2**	215.0	1.074
3	**Ctrl_Xp22**	160.5	0.997
4	**Ctrl_Xq11**	335.9	1.044
5	**Ctrl_Xq21**	371.3	1.002
6	**Ctrl_Xq23**	127.1	0.982
7	**Ctrl_Xq26**	257.0	1.005
8	**Ctrl_Xq27**	301.2	0.971
9	LCR1	309.8	1.002
10	LCR2	354.9	1.079
11	LW/MW_ex4_c607T	174.2	1.518
12	LW/MW_ex6_1083A	153.8	1.440
13	LW_ex1_c.-77A	167.8	1.081
14	LW_ex2_c.300A	188.7	1.020
15	LW_ex3_c.457C	225.1	1.520
16	LW_ex4_c.706A	201.8	0.964
17	LW_ex5_c.830A	237.4	0.992
18	LW_ex5_c.926A	319.0	1.034
19	MW_ex1_c.-62A	147.6	2.006
20	MW_ex2_c.300G	195.4	1.940
21	MW_ex3_c.457A	231.4	1.561
22	MW_ex4_c.706G	208.0	1.963
23	MW_ex5_c.830T	243.5	1.921
24	MW_ex5_c.926T	325.1	1.816

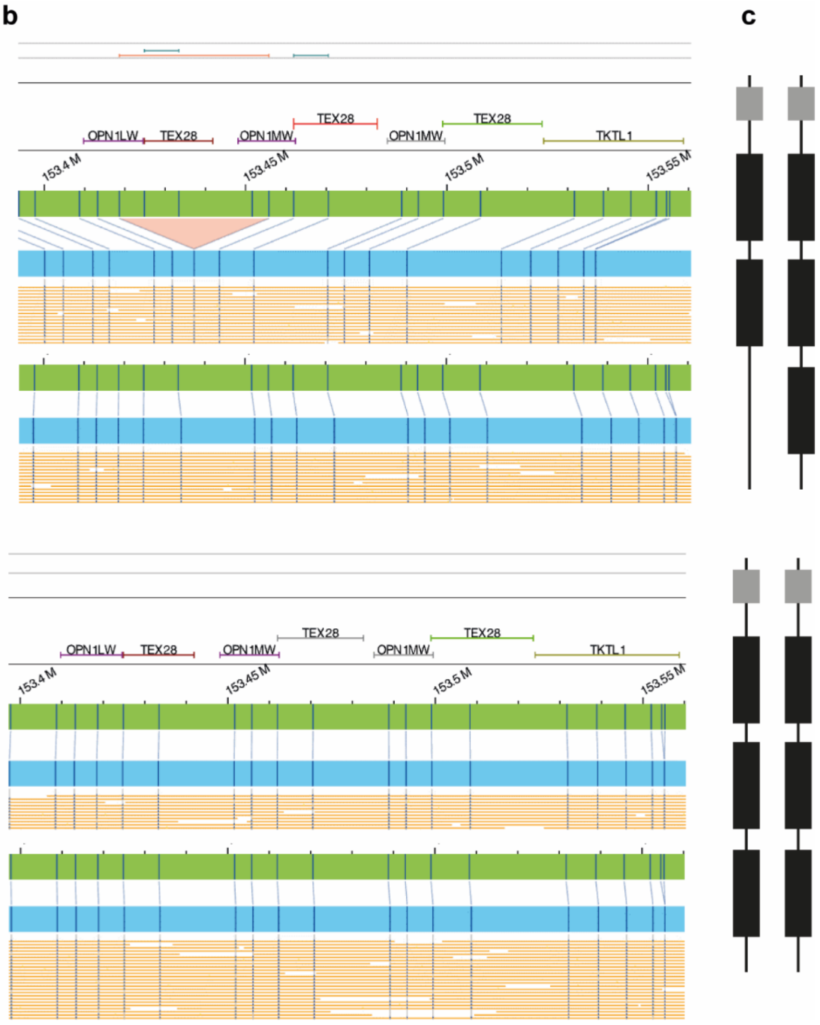

Supplementary Figure 2 Copy number analysis of the OPN1LW/OPN1MW cluster via MLPA and OGM. a Results of the MLPA are stated in the depicted table. The column "height ratio" refers to the ratio that is calculated for each probe of the sample compared to a reference with 1 LCR, 1 *OPN1LW* and 1 *OPN1MW* gene copy. A ratio of ~1 means the sample has the same copy number as the reference sample, a ratio of two means the sample has twice the amount of copies compared to the reference, etc. It is of importance that probe LM/MW_ex4_c607T and LM/MW_ex6_1063A target both *OPN1LW* and *OPN1MW* thus the reference copy number for these probes is 2. Moreover for female samples the ratio for all probes has to be multiplied by 2, as women have two X-chromosomes. In control sample S3.1 the ratios of all probes targeting only *OPN1LW* are ~1, the ratios of all probes targeting only *OPN1MW* are ~0 and the probes LM/MW_ex4_c607T and

LM/MW_ex6_1063A targeting both *OPN1LW* and *OPN1MW* are ~0.5, indicating that a single *OPN1LW/OPN1MW* gene copy, most likely a *OPN1LW* gene, is present in this sample. In sample S3.2 ratios of the probes targeting *OPN1LW* and *OPN1MW* are either ~0, ~1 or ~2, indicating the presence of two *OPN1LW/OPN1MW* gene copies and possibly the *OPN1LW* gene is a hybrid gene. In sample S3.3 ratios of most of the targets to *OPN1LW* are ~1 and the ratios of targets to *OPN1MW* are ~2, indicating the presence of three *OPN1LW/OPN1MW* gene copies. In sample S3.4 ratios of probes targeting *OPN1LW* are either 0.5 or 1 and ratios of probes targeting OPN1MW are either ~1.5 or ~2, as this is a female sample, the ratio has to be multiplied by 2. Therefore the MLPA indicates the presence of five *OPN1LW/OPN1MW* gene copies, and possibly a hybrid *OPN1LW* gene is present. In GIAB sample NA12878 ratios of probes targeting *OPN1LW* are ~1 and probes targeting *OPN1MW* are ~2, as this is a female sample, the ratio has to be multiplied by 2. Therefore the MLPA indicates the presence of two *OPN1LW* and four *OPN1MW* gene copies, in total six *OPN1LW/OPN1MW* gene copies. **b** Results of OGM. Green bars represent the genome maps of the reference (here: GRCh37/hg19), blue bars represent genome maps of the respective samples. Small vertical bars within the genome maps represent fluorescently marked labels (GAATTC). By comparing the labels of the genome map of the sample of interest to the labels in the reference, structural variants and copy number changes can be detected. The single molecules forming the genome maps are shown underneath in orange. The reference GRCh37/hg19 includes 1 *OPN1LW* and 2 *OPN1MW* gene copies. As OGM is not a sequencing technique, OGM can't determine whether a opsin gene copy is a *OPN1LW* or *OPN1MW* gene copy and therefore only the total number of *OPN1LW/OPN1MW* gene copies has been stated. In the male sample S3.1 only 1 allele is shown, in which the 2 *OPN1MW* copies are not present (depicted by orange triangle). Accordingly, this sample carries 1 *OPN1LW/OPN1MW* gene copy. The male sample S3.2 shows 2 *OPN1LW/OPN1MW* gene copies. Male sample S3.3 carries 3 *OPN1LW/OPN1MW* gene copies. Female sample S3.4 shows 2 *OPN1LW/OPN1MW* gene copies on one allele (upper genome maps) and 3 *OPN1LW/OPN1MW* gene copies on the other allele (lower genome maps). GIAB sample NA12878 is a female as well, with 3

OPN1LW/OPN1MW gene copies on each allele. **c** Schematic overview of the number of *OPN1LW/OPN1MW* gene copies determined for each sample by both MLPA and OGM. As MLPA and OGM only give an indication whether the detected gene copy is either *OPN1LW* or *OPN1MW*, all detected *OPN1LW/OPN1MW* gene copies are depicted in black, the LCR is depicted in grey. The distribution of the number of *OPN1LW/OPN1MW* gene copies on each of the two alleles in the female samples S3.4 and NA12878 was solely based on the data of OGM, as MLPA can only determine the copy number of both alleles together.

Supplementary Figure 3

a LCR

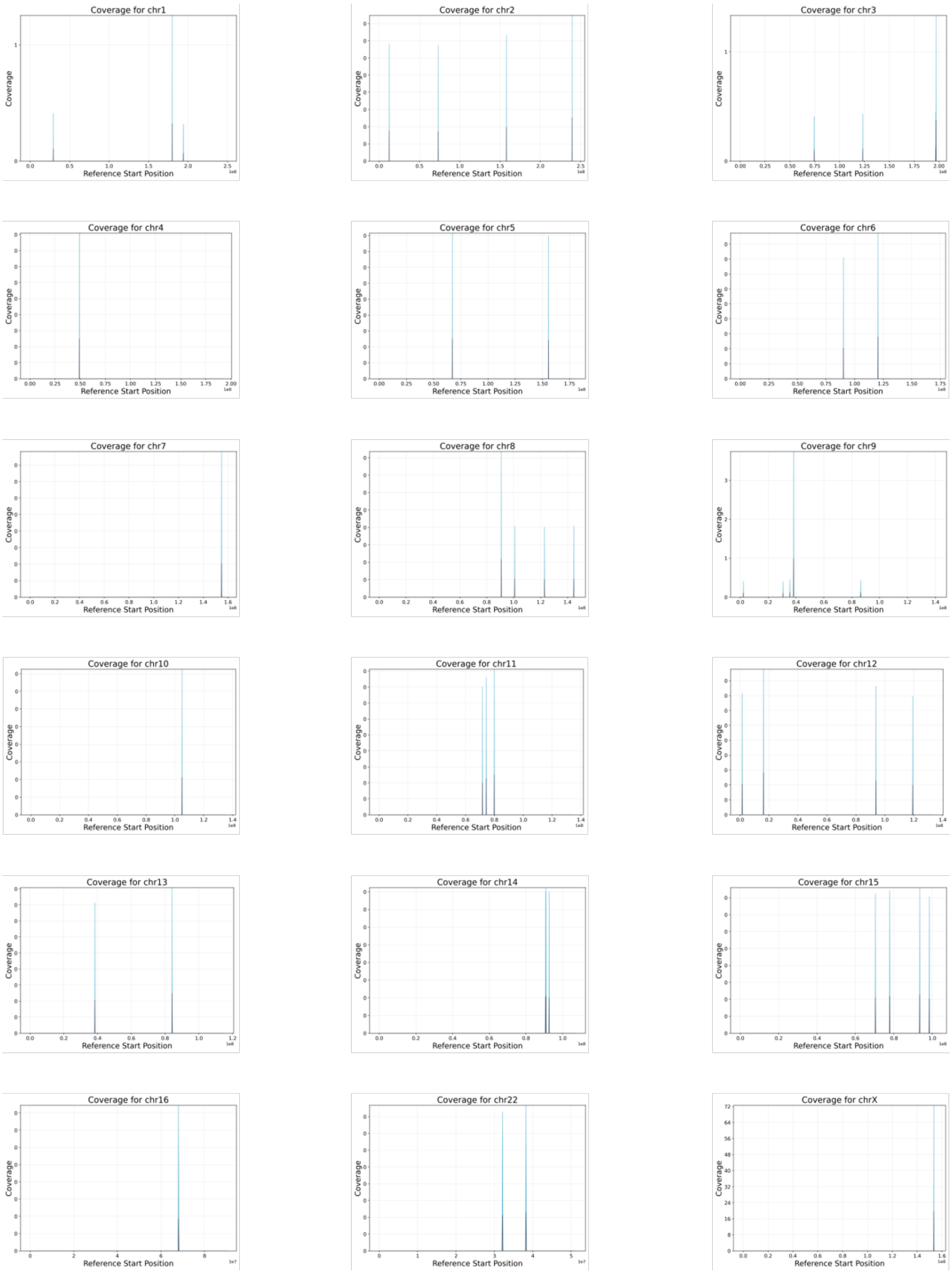

b First opsin gene of the *OPN1LW/OPN1MW* gene cluster

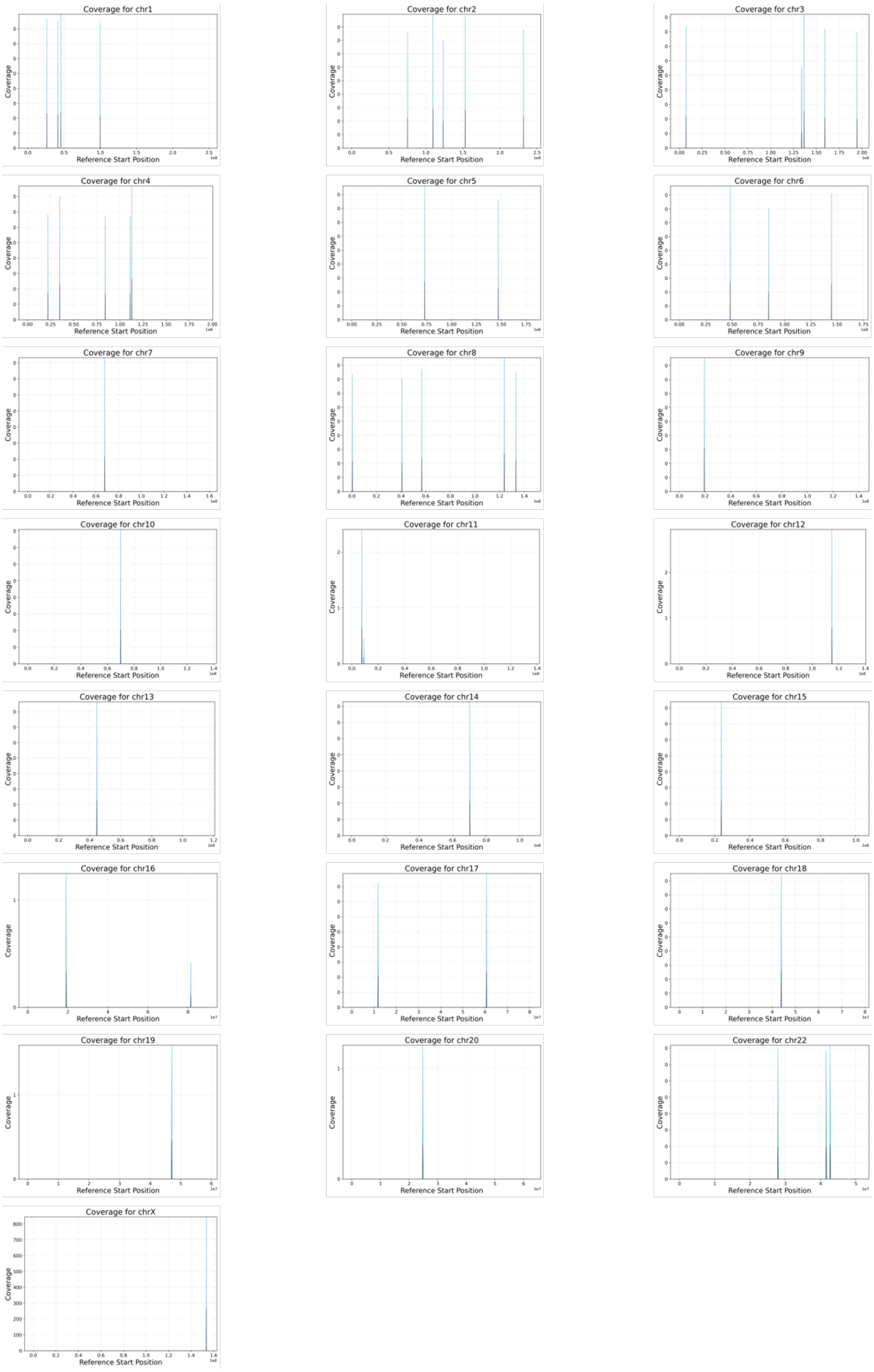

c Second (and consecutive) opsin gene(s) of the *OPN1LW/OPN1MW* gene cluster

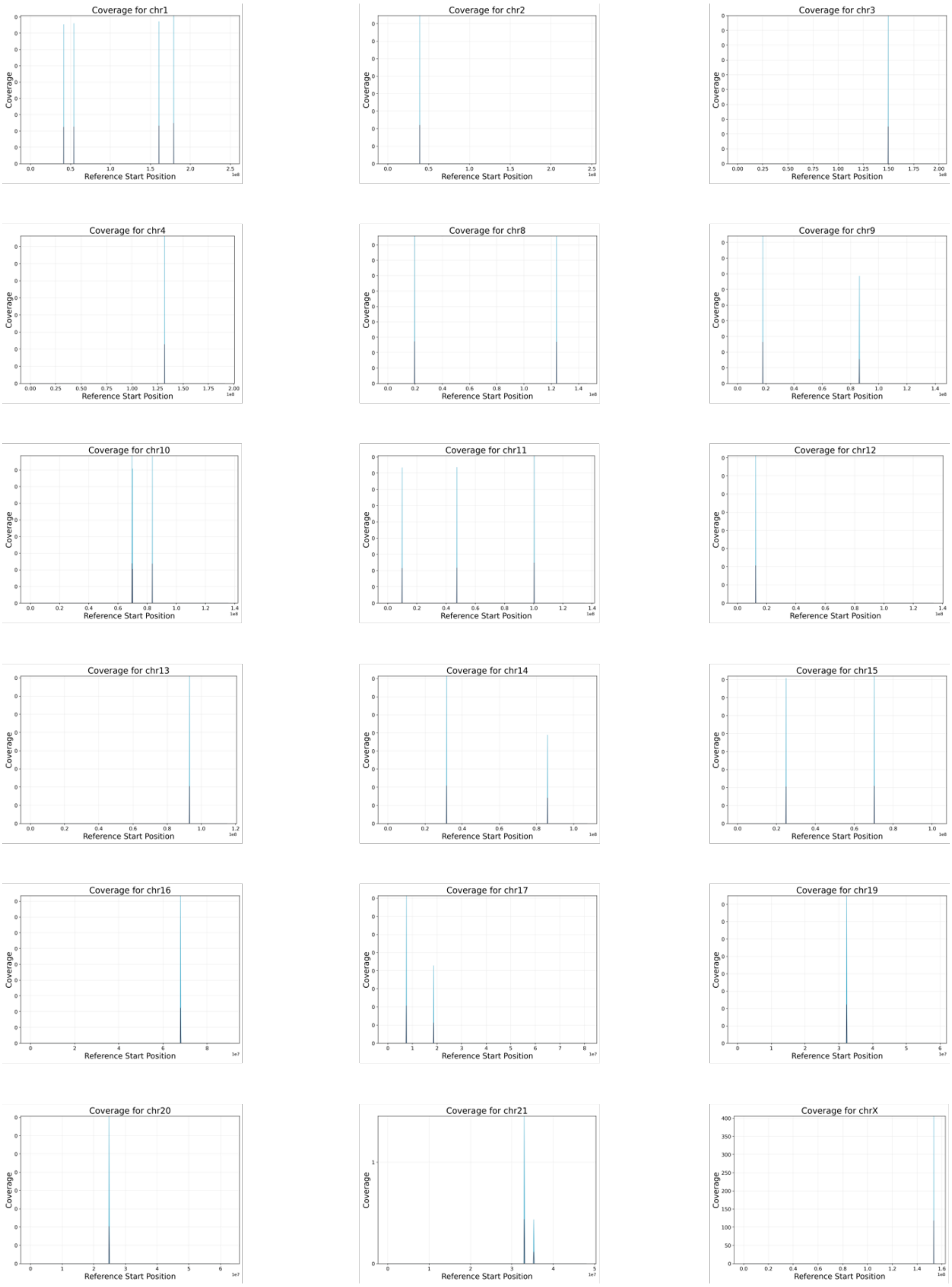

d Last opsin gene of the *OPN1LW/OPN1MW* gene cluster

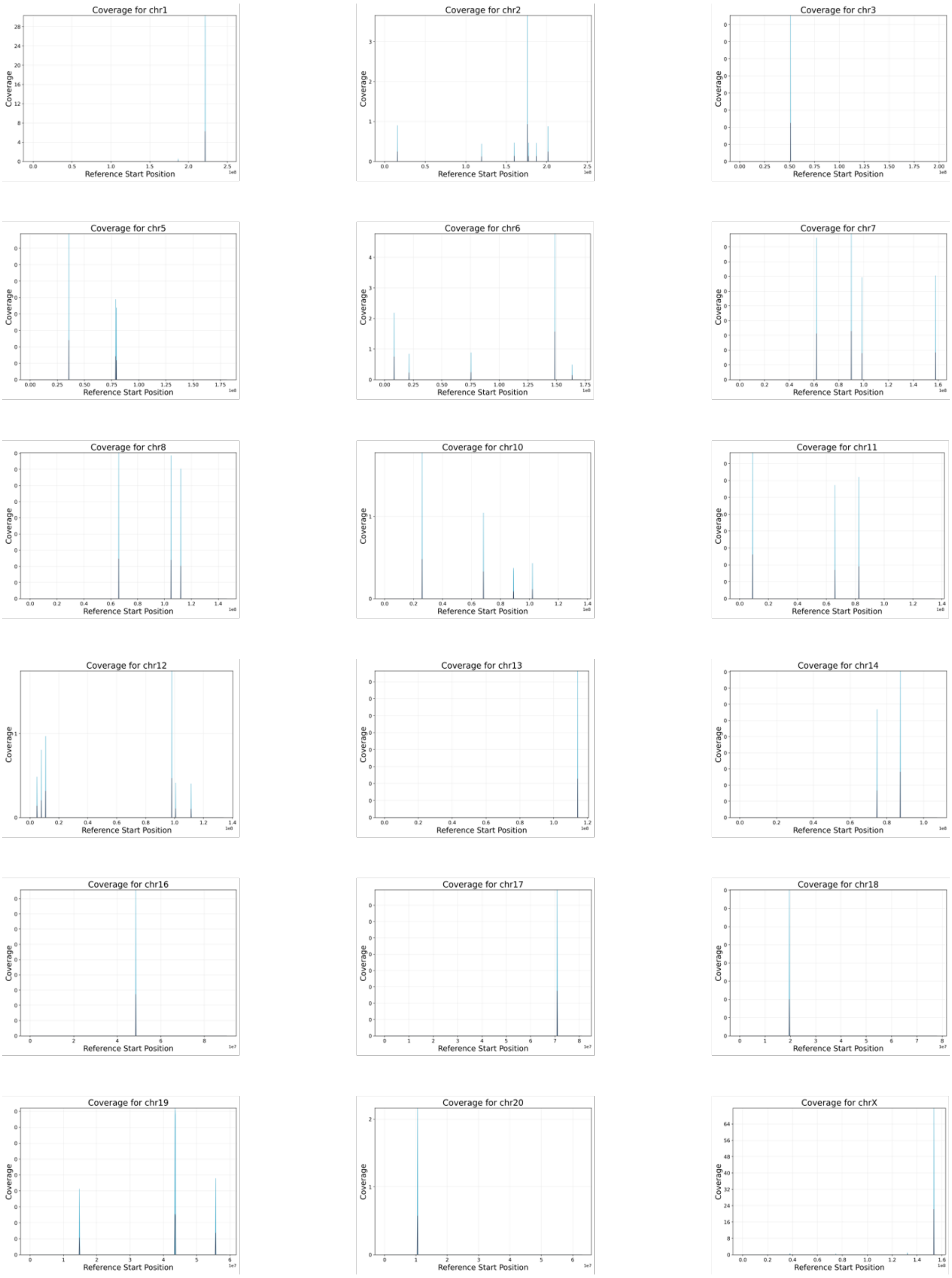

Supplementary Figure 3 Coverage across chromosomes: Thumbnails of the results of genome wide mapping of reads for each of the four amplicons. Representative thumbnails showing mapping of CCS reads larger than 10kb for one of the male control samples, provided by SMRTLink. Only chromosomes to which reads are mapping are shown. The scale on the Y-axis is indicating the depth of coverage, the position given on the X-axis is providing the location on the respective chromosome. **a** Thumbnails for LCR. Several chromosomes show some background noise. Higher coverage (appr. 70x) is only seen on chromosome X. **b** Thumbnails for the amplicon of the first gene of the *OPN1LW/OPN1MW* gene cluster. Several chromosomes show some background noise. High coverage (appr. 800x) is only seen on chromosome X. **c** Thumbnails for the amplicon of second (and consecutive) gene(s) of the *OPN1LW/OPN1MW* gene cluster. Several chromosomes show some background noise. Higher coverage (appr. 400x) is only seen on chromosome X. **d** Thumbnails for the amplicon of the last gene of the *OPN1LW/OPN1MW* gene cluster. Several chromosomes show some background noise. Unspecific coverage of around 30x is seen on chromosome 1, the specific region on chromosome X shows a coverage of around 70x.

Supplementary Table 1 Genotyping results of patients affected with visual impairment with color deficiencies

Provided in Excel sheet format

Supplementary Table 2 Carrier analysis in females for the OPN1LW/OPN1MW gene cluster

Provided in Excel sheet format

Supplementary Table 3 Quality parameters of long-read sequencing of the amplicons

Provided in Excel sheet format

Supplementary Table 4 Probe sequences of the MLPA of the OPN1LW/OPN1MW gene cluster

Probe number	Reference probe	Gene	Target	Left probe sequence	Right probe sequence
02634-L02101	True	*OTC*		GTCCTTAGGCATGATTTTTGAGAAAAGAAGTA	CTCGAACAAGATTGTCTACAGAAACAGGTAAGTCCACTGCCAAA
04994-L04380	True	*GPC3*		GAACTTGAGCTCCATACTTGCAGACTGAAG	CAGCTGTTCCATGTTCAATCGTGCTGTTAGTTGGTATTTT
06457-L06570	True	*CDKL5*		TCAGTGAGAATTTCTTCCTTCAGACGGTT	TTGGATCTTACTGCACAGCTTTCTGAGAAGTTCTTTTGGTGCCA
06792-L06384	True	*F9*		CCTATCTCAAAGATGGAGATCAGTGTGAGT	CCAATCCATGTTTAAATGGCGGCAGTTGCAAGGATGACAT
07479-L07136	True	*ATP7A*		CATCAGAGGCTCTTGCAAAGTTAATTTCACTAC	AAGCTACAGAAGCAACTATTGTAACTCTTGATTCTGATAATATCCTCCT
07648-L07354	True	*FGD1*		GAGGAGGAGGAGGAAGAGAAGGACAGAGAA	ATCCCAGTGCCCCTGATGGAGAGACAGGAGTCTGTGG
13917-L18702	True	*AGTR2*		CTTGCATTATGGCTTTCCCACCTGA	GAAATATGCCCAATGGTCAGCTGGGATTGCCTTAATGA
14801-L13868	True	*AMER1*		GCTCCAAGAAAGGTCTCAGCAAGAGCA	AGACCCACGATGGCCTGAGTGAAGCAGCCCATGG
21601-L30183	False	*OPN1LW*	LCR/c.-3531A	CAGGTTCTGCACCTTCACCGGCTCTGCTAATT	GATGATTAGGGCTAACAGAAGATCAAGTCCCATTTTAGCCT
21612-L30197	False	*OPN1LW*	LCR/c.-3930A	CTCCCGCGTGGAGTCATTTAGGAGTAGTCGCATTA	GAGACAAGTCCAACATCTAATCTTCCACCCTGGCC
21605-L31767	False	*OPN1LW*	c.-77A	CCTTAAGGTGAAGAGGCCCGGGCTGATCCCACA	GGCCAGTATAAAGCGCCGTGACCCTCAGGTGATGCGCCAG
21609-L30261	False	*OPN1LW*	c.300A	GGATCCTGGTGAACCTGGCGGTCGCTGACCTA	GCAGAGACCGTCATCGCCAGCACTATCAGCGTTG
21613-L30201	False	*OPN1LW*	c.457C	CTGGTCTCTAGCCATCATTTCCTGGGAGAGGTGGC	TGGTGGTCTGCAAGCCCTTTGGCAATGTGAGATTTGAT
21611-L30198	False	*OPN1LW*	c.706A	CACCTGCTGCATCATCCCACTCGCTATCATCA	TGCTCTGCTACCTCCAAGTGTGGCTGGCCATCCGAGCGGTGGCA
21615-L30200	False	*OPN1LW*	c.830A	CGCATGGTGGTGGTGATGATCTTTGCGTA	CTGCTTCTGCTGGGGACCATACGCCTTCTTCGCAT
21602-L30184	False	*OPN1LW*	c.926A	CTACGCCTTCCACCCTTTGATGGCTGCCCTGCCGGCCTA	CTTTGCCAAAAGTGCCACTATCTACAACCCCGTTATCTATGTC
21606-L31768	False	*OPN1LW* and *OPN1MW*	c.607T	TCAGTACGAGCTGCCGCTGAACACGTCTGGGCCGCA	TGAAGTCTTCAGGCCGTGGGGCCAGTACCTGGAGAGA
21608-L30190	False	*OPN1LW* and *OPN1MW*	c.1083A	TCCAAAACGGAGGTCTCATCTGTGTCCTCGGTA	TCGCCTGCATGAGGTCTGCCTCCTACCCATCCCG
21603-L30185	False	*OPN1MW*	c.-62A	TAGAGGCCCGGGCTGATCCCACTGGCCGGTATAAAGCA	CCGTGACCCTCAGGTGACGCACCAGGGCCGGCTGC
21609-L30193	False	*OPN1MW*	c.300G	CCTTGAAGGATCCTGGTGAACCTGGCGGTCGCTGACCTG	GCAGAGACCGTCATCGCCAGCACTATCAGCGTTG
21613-L30199	False	*OPN1MW*	c.457A	CCTGAACTGGTCTCTGGCCATCATTTCCTGGGAGAGATGGA	TGGTGGTCTGCAAGCCCTTTGGCAATGTGAGATTTGAT
21611-L30196	False	*OPN1MW*	c.706G	CGATTGCACCTGCTGCATCACCCCACTCAGCATCATCG	TGCTCTGCTACCTCCAAGTGTGGCTGGCCATCCGAGCGGTGGCA
21615-L30202	False	*OPN1MW*	c.830T	CGTAACCGCATGGTGGTGGTGATGGTCCTGGCATT	CTGCTTCTGCTGGGGACCATACGCCTTCTTCGCAT
21602-L30194	False	*OPN1MW*	c.926T	CATGTACTACCCCTTCCACCCTTTGATGGCTGCCCTGCCGGCCTT	CTTTGCCAAAAGTGCCACTATCTACAACCCCGTTATCTATGTC

Supplementary Table 5 Quality parameters of OGM

Sample	Data collected (Gb)	N50 >=150 kbp (Mbp)	N50 >= 20kbp (Mbp)	Avg label density (per 100kbp)	Avg. map rate %	effective coverage(X)
Control S3.1	844	0.282	0.209	15.2	88.4%	231.6
Control S3.2	809	0.336	0.249	17.5	84.2%	212.7
Control S3.3	859	0.325	0.296	15.3	94.2%	250.2
Control S3.4	808	0.284	0.207	15.2	91.9%	230.5
GIAB sample NA12878	329	0.222	0.119	15.6	83.0%	85.8
